# Potential of North American Acorns as an Underutilized Food Source: Morphology, Nutritional Composition and Content of Bioactive Compounds in *Quercus virginiana* Acorns of Different Natural Populations

**DOI:** 10.3390/molecules31091438

**Published:** 2026-04-27

**Authors:** José Valero-Galván, Oscar A. Muñoz-Bernal, Raquel González-Fernández, Jesús V. Jorrín-Novo, Laura A. De la Rosa

**Affiliations:** 1 Departamento de Ciencias Químico-Biológicas, Instituto de Ciencias Biomédicas, Universidad Autónoma de Ciudad Juárez, Av. Plutarco Elías Calles #1210 Fovissste Chamizal, Ciudad Juárez, Chihuahua C.P. 32310, Mexico; jose.valero@uacj.mx (J.V.-G.); adrian.munoz@uacj.mx (O.A.M.-B.); raquel.gonzalez@uacj.mx (R.G.-F.); 2 Secretaría de Ciencia, Humanidades, Tecnología e Innovación (SECIHTI), Insurgentes Sur 1582, Col. Crédito Constructor, Alcaldía Benito Juárez, Ciudad de México C.P. 03940, Mexico; 3 Agroforestry and Plant Biochemistry, Proteomics, and Systems Biology, Department of Biochemistry and Molecular Biology, ETSAM, University of Cordoba, UCO-CeiA3, 14014 Cordoba, Spain; bf1jonoj@uco.es

**Keywords:** *Quercus virginiana*, non-conventional foods, seed morphology, nutrients, antioxidants, phytochemicals

## Abstract

*Quercus* acorns have been part of animal or human diets; however, their nutritional potential depends on morphological and chemical characteristics highly influenced by genetic and geographical factors. Research on the *Quercus* genus has focused on Asian and European species overlooking the American taxa. Therefore, this study aimed to evaluate the morphological and nutritional characteristics, and the content and profile of bioactive compounds of acorns from four populations of the American species *Quercus virginiana* from the state of Chihuahua, Mexico. Discriminant function analysis showed a well-established group formed by the two southern populations (CH), while the two northern populations were separated into different groups (CJA and CJB). CH populations showed smaller seeds (1.4 g, 2.0 cm length) and higher starch (57–58%), oleic acid (65–70%), phenolic compounds (78–176 mg GAE/g), flavonoids (29–37 mg CE/g), and antioxidant activity (278–282 μmol TE/g). Acorns from the CJA population were the largest (2.3 g, 2.4 cm length) and displayed the highest protein content (7.0%). Acorns from the CJB population showed the highest values for ash (2.2%), sugars (13.8%), palmitic and linoleic acids (19.1%), and condensed tannins (0.26 CE/g). Fourteen polyphenolic compounds were identified: twelve hydrolysable tannins; one hydroxycinnamic acid, and one flavonol. These variations reflected the impact of local climatic and geographic conditions and may influence the potential use of *Quercus* acorns in sustainable agriculture and food development.

## 1. Introduction

The genus *Quercus* (oaks; Fagaceae) is one of the largest and most important plant taxonomic groups in Mexico, with 125–161 species reported [1]. The states with the highest diversity are Oaxaca, Nuevo León, Jalisco, Chihuahua, and Veracruz [1]. In these regions, *Quercus* species are a valuable forest resource that provides excellent quality wood, as well as other non-timber products [2]. Acorns, the seeds of the *Quercus* genus, have served as an important resource for rural economies since ancient times, primarily as a component of animal feed [3,4]. However, recent investigations have highlighted the significant content of bioactive compounds (including phenolic compounds, fiber, and unsaturated fatty acids) in acorns [5,6]. Among phenolic compounds, the most common in acorns are α and γ tocopherols in the oil fraction and gallic and ellagic acid derivatives (including gallotannins and ellagitannins) in the flour [3,7], although flavonoids such as catechin, epicatechin and quercetin have also been identified in some species [8,9]. The consumption of these compounds is related to a reduced risk of cancer, cardiovascular diseases, cataracts, and brain dysfunction, so there has been a growing interest in the use of acorns as raw materials for the development of human foods [6]. In addition, an increase in acorn production to use as a human food ingredient can have a positive impact on the social and economic levels, aiding in sustainable farm development and promoting environmentally friendly industrial processes [4].

On the other hand, several studies have demonstrated a high variation in the morphometric characteristics [8,10] and chemical composition of the acorn from different *Quercus* species [8,11]. Among the factors that most influence these variations are intraspecific variability, the species provenance and its climatic conditions, as well as the maturity state of the acorn [11,12]. However, these studies have been carried out on *Quercus* species from the Asian and European continents, leaving aside the American species.

*Q. virginiana* is a species widely distributed in the north of the American continent, it presents various growth forms, ranging from a rhizomatous shrub to a large tree reaching up to 24 m in height, with a dense spreading crown and a trunk buttressed at the base. The acorns are annual, dark brown to nearly black, and narrowly oblong, varying from 16 to 25 mm in length. In Mexico, this species propagates in the northeast of the country including the states of Chihuahua, Coahuila, Nuevo Leon, and Tamaulipas. In recent decades, *Q. virginiana* has been transplanted into the urban ecosystem and has been well established in cities in the north of Mexico because they are trees with the ability to withstand the wind and salinity of soils. The acorn of *Q. virginiana* has been analyzed and compared to that of Mediterranean *Quercus* species. The acorn of *Q. virginiana* presented the lowest weight, length, diameter, coat weight, and megagametophyte weight with respect to the acorns of *Q. ilex*, *Q. suber*, and *Q. coccifera*; however, it showed the highest lipids, energy, and digestibility, and the lowest starch and carbohydrate contents [11]. Only two other studies have been found that describe the nutritional and chemical characteristics of *Q. virginiana* acorns. A study on American oak species reported an acorn nutrient composition of 4.6% protein, 5.8% fat, and 18.6% fiber [13]. Finally, an analysis of *Q. virginiana* seeds from different provenances in China indicated considerable variation in fatty acid profiles, with oleic acid ranging from 42.6 to 58.7%, palmitic acid from 18.4 to 25.5%, linoleic acid from 18.9 to 23.3%, and stearic acid from 1.4 to 1.9% among the different populations [14]. Therefore, information on population-level variation in the morphological characteristics and nutritional composition of *Q. virginiana* is lacking for Mexican populations. Moreover, to our knowledge, the identification of individual phenolic phytochemicals has not been performed in this species.

To address this knowledge gap, four *Q. virginiana* populations from the northern Mexican state of Chihuahua were studied. Two populations were selected from the north of the state and two from the south, to represent latitudinal and environmental variability within the state of Chihuahua and to capture potential ecological differentiation across the *Q. virginiana* distribution range in this North American region. We hypothesized that the environmental contrasts between northern and southern Chihuahua would be reflected in variation in acorn morphometric features and chemical composition among these populations.

Hence, this study aimed to evaluate the seed morphometry, nutritional composition, profile and content of phenolic compounds and antioxidant activity of four populations of *Q. virginiana* from the state of Chihuahua in the north of Mexico. Ultimately, the characterization of *Q. virginiana* populations in northern Mexico, and their intra-population variation, can support the valorization of acorns as ingredients for functional foods and provide valuable information for conservation, restoration, and seed source selection programs in this region.

## 2. Results and Discussion

### 2.1. Acorn Morphometry Analysis

Statistically significant differences were observed in the acorn morphometric traits among populations (*p* ≤ 0.05). Both CJ populations, located in the most northern part of the state, had the highest values for mass, length, width, area, perimeter, seed coat weight, megagametophyte mass, length, width, area, and perimeter, whereas the two populations located in Chihuahua City (CHA and CHB) had the lowest values (Table 1).

**Figure 1:**
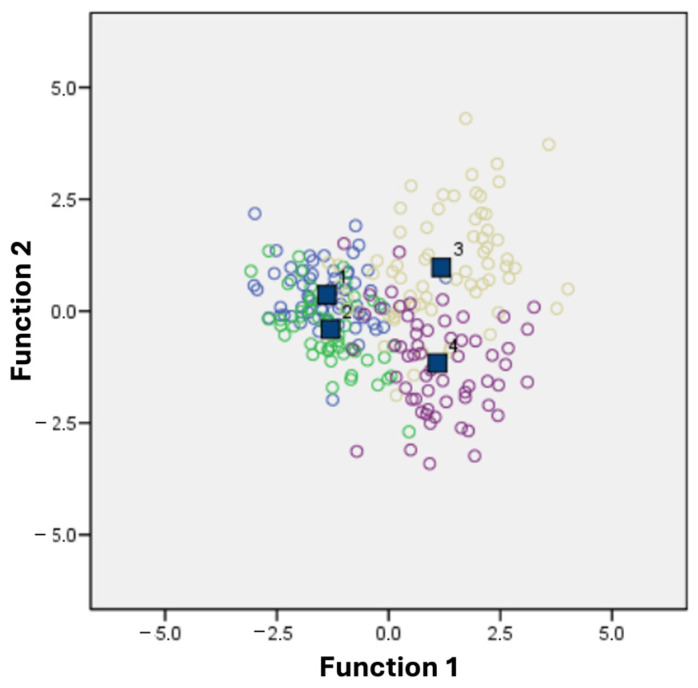
A combination of the canonical scores of the first two canonical discriminant functions of seed morphometry analysis of the four populations of *Quercus virginiana*. CHA (1); CHB (2); CJA (3); CJB (4).

Canonical discriminant analysis of the morphological variables of acorns showed that the first two functions accounted for 90.9% of the total variation. The first function explained 62.9% of the total variation, and the variables that correlated positively with this function were seed width (r = 0.789), seed mass (r = 0.675), and megagametophyte area (r = 0.668). The value of the second function represented 28.0% of the total variation, and no variables were correlated with this function. To evaluate the classification performance and stability of the discriminant functions, several diagnostic metrics were examined. The original classification matrix indicated that 73.8% of cases were correctly classified, with groupwise accuracy ranging from 66.0% to 78.3%. Leave-one-out crossvalidation (LOOCV) yielded a similar accuracy (72.1%), with groupwise values ranging from 64.0% to 76.7%, indicating that the model is robust and not overly dependent on the training sample. The dispersion diagram of the two discriminant functions showed three well-established groups. The first one was formed by the CHA (1) and CHA (2) populations; the second one was formed by the CJA (3) population which was distributed towards the upper right part of the diagram, and finally the third group was formed by the CJB (4) population which was distributed towards the lower right part of the diagram (Figure 1).

These differences in the values of the seed morphometric traits could be associated with the ecological environment in which the population grew, and the genotypic variability. To investigate if the climatic and geographical variables of the growth location were correlated with the seed morphometric traits, a Pearson correlation analysis was conducted (Table S1 in Supplementary Materials).

**Figure 2:**
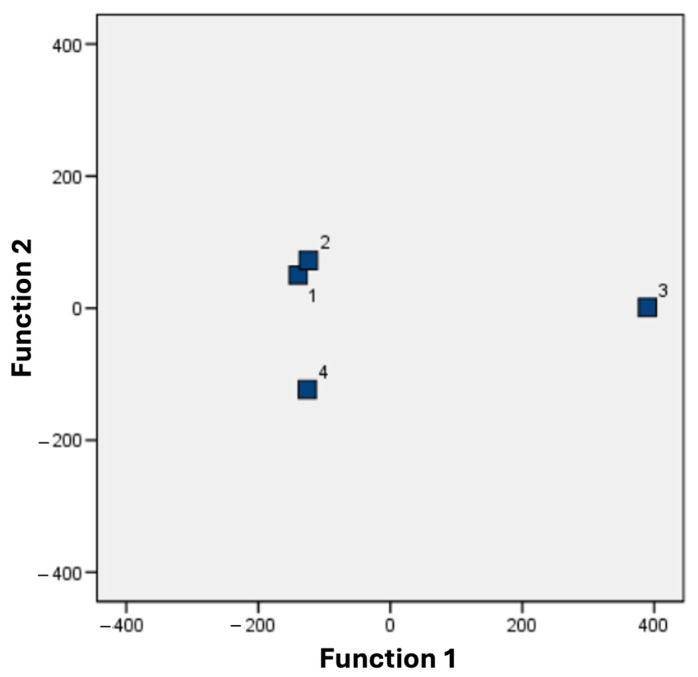
A combination of canonical scores of the first two canonical discriminant functions of seed chemical and phytochemical analyses of four populations of *Quercus virginiana*. CHA (1); CHB (2); CJA (3); CJB (4).

Interestingly, the acorns from the two populations that grew in the southern part of the state presented the smallest values in weight and size, whereas the acorns collected from the northern populations included the widest and longest acorns. In fact, there was a positive correlation of latitude with seed length, width, area, and perimeter (Table S1). These results were like those found for *Q. ilex* and *Q. suber* seeds, where the weight and size were positively correlated with the latitude of the collection sites [12,15]. A positive correlation between seed size and latitude has also been observed in other American *Quercus* species [16]. In our study, altitude was negatively correlated with the seed length, width, area, and perimeter, indicating that the populations growing at the highest altitudes presented the smallest and thinnest acorns (Table 2, Table S1 and Figure 2). These results were like those observed in *Q. ilex* and *Q. glauca* seeds, where weight and size were negatively correlated with the altitude [12,17]. Climatic variables such as lower temperatures, lower rainfall, and high seasonal variation in rainfall have been related to seed speedy germination, seed dispersal at higher moisture contents and water potentials, large embryonic axes, and rapid drying. Higher temperatures, higher rainfall, and low seasonal variation in rainfall have been associated with large seed mass, thick pericarp, and higher optimal temperatures for acorn germination [18]. In contrast, our results showed that annual precipitation was negatively correlated with seed weight and size (Table S1), but this could also be related to the effect of latitude, since the largest seeds were found in the CJ sites which are in the northern part of the state and present the lowest annual precipitation. It is believed that there are conflicting selection pressures on acorn size. For example, the largest size and mass of acorns increased the root and shoot ratios, seedling height, and seedling growth [15,17,19]; however, larger acorns may suffer higher predation rates, and bird dispersers usually prefer smaller acorns [20,21]. From the perspective of food production, bigger acorns may be preferred over smaller ones, which makes the northern populations of *Q. virginiana* interesting as a potential food resource, despite the low rainfall of the region. Overall, these patterns indicate that the morphometric variations observed reflect not only spatial differentiation but also ecological and functional adaptations to environmental gradients. This suggests that *Q. virginiana* populations may experience distinct selection pressures affecting the size and shape of their seeds.

### 2.2. Nutritional Composition and Content of Bioactive Compounds

There were statistically significant differences (*p* ≤ 0.05) in the contents of nutrients and bioactive compounds of *Q. virginiana* acorns among the four populations examined. In nutrients, CHA presented the lowest values for ash, protein, fat, sugar, digestibility, energy, palmitic, and linoleic acids, and the highest values of carbohydrates, starch and oleic acid. CJA presented the lowest values for carbohydrates and starch, and the highest protein, fiber, digestibility, and energy. CJB presented the lowest values for stearic and oleic acids, and the highest contents of ash, sugar, palmitic, and linoleic acids. CHB presented the highest contents of stearic acid (Table 2).

The contents of bioactive compounds showed statistically significant differences at the population level in phenols, flavonoids, condensed tannins, and antioxidant activity determined by FRAP (*p* ≤ 0.05). No statistically significant differences were observed in carotenoid content and antioxidant activity determined by DPPH. The results showed that CH populations presented the highest values of phenolic compounds, flavonoids, and antioxidant activity determined by the FRAP assay. The content of phenolic compounds in the CHB population was considerably higher (2–3 times) compared to all the other populations, and CJB had the highest condensed tannin content (Table 2).

The water content was similar among the four populations in this study; these results were like those determined in a previous study of *Q. virginiana* seeds from a population located in Ciudad Juárez (CJ), Chihuahua, Mexico (73%) [11]. Ash content, which essentially represents the mineral content of foods, varied between 1.8% and 2.2%. These values were slightly higher than those observed in the previous study of Valero-Galván et al. [11] (1.6%). Acorns are recognized as good sources of some minerals, especially K, Mg, Ca and P [6,22], which are important micronutrients in the human diet.

Acorns are considered an underutilized source of nutrients, primarily providing carbohydrates with moderate amounts of protein and fat. In our study, the carbohydrate content varied between 79.3 and 82.5%, protein between 4.3 and 7.0%, and fat between 11.3 and 12.7%, in the acorns of four *Q. virginiana* populations from the north of Mexico. These values were like those observed in a previous study of *Q. virginiana* from the same region [11] and are also in the range reported for other *Quercus* species [6].

The primary carbohydrate found in acorns is starch, which is a complex carbohydrate and a major source of energy in the human diet. Acorns also contain lesser amounts of simple sugars, such as glucose and fructose, and dietary fiber. Fiber is a type of carbohydrate that cannot be digested by the human body, so it is not an energy source, but it plays a significant role in digestive and overall health. In our study, starch content ranged from 53.4% to 58.8%, and simple sugar content varied between 8.1% and 13.8%. These values are like those previously reported in *Q. virginiana* and other species [6,11]. Our results show that fiber content varied between 2.4 and 2.5%, values similar to those reported by Valero-Galván et al. [11] but considerably lower than the fiber content reported in *Quercus rotundifolia* (11.4%) and other *Quercus* species which are considered rich in dietary fiber [22].

Acorns, while not as fat-rich as other nuts, do contain various fatty acids that contribute to their positive nutritional profile. The predominant monounsaturated fatty acid in acorns is oleic acid, which is known for its benefits in reducing LDL-cholesterol levels and potentially increasing HDL-cholesterol levels, thus contributing to heart health [23,24]. Our results confirmed oleic acid as the major fatty acid in acorn. Oleic acid content varied from 58.3 to 70.8% in the four *Q. virginiana* populations from the north of Mexico. These values were higher than those observed in *Q. virginiana* seeds in populations from three regions of China (42.6–58.7%) [14], but like those observed in Mexican *Q. virginiana* seeds [11]. Palmitic acid was the second most abundant fatty acid in *Q. virginiana* acorns, although in the CJB population, palmitic and linoleic acid contents were equal. Palmitic acid is a saturated fatty acid found in many plants and animals, and, like other saturated fats, it is a dense source of energy. In our study, palmitic acid content varied from 14.6 to 19.1%; these values were lower than those observed in *Q. virginiana* seeds in populations from three regions of China (18.4–25.5%) [14], but like those observed in Mexican *Q. virginiana* seeds [11]. Linoleic acid is a polyunsaturated fatty acid and an essential omega-6 fatty acid, meaning that the human body cannot synthesize it and must be obtained through diet. Acorns contain important levels of linoleic acid, which contributes to their positive nutritional profile. Our results showed a variation in linoleic acid content from 12.4 to 19.1%, which was lower than that observed in *Q. virginiana* seeds from populations located in three regions of China (18.9–23.3%) [14], but similar to that observed in Mexican *Q. virginiana* [11]. Interestingly, the two CJ populations, located in the northern part of the sampling region, showed significantly higher levels of linoleic and palmitic acids, and lower levels of oleic acid (Table 2). Stearic acid, a saturated fatty acid, was also present in acorns, although at lower levels than oleic, linoleic, and palmitic acids. In our results, stearic acid content ranged from 3.0 to 4.3%, which was higher than that observed in *Q. virginiana* seeds from populations located in the three regions of China (1.4–1.9%) [14].

Acorns are not a particularly rich source of carotenoids, and their content can vary depending on the tree species and environmental factors like sunlight exposure, soil quality, etc. [4]. The major carotenoids identified in acorns are β-carotene and lycopene, with contents ranging from 47.3 to 131.2 mg/100 g for β-carotene and 7.9 to 18.3 mg/100 g for lycopene, in *Q. faginea*, *Q. ilex,* and *Q. suber* [6]. In our results, total carotenoid content varied from 1.1 to 5.1 mg/g, equivalent to 110 to 510 mg/100 g (Table 2).

Phenolic compounds are the most abundant group of bioactive compounds in the plant kingdom; they are also widely recognized for their antioxidant activity, and their role in the prevention of numerous noncommunicable, chronic or degenerative diseases [25]. Phenolic compounds possess great structural variability, but the most common groups in plants are flavonoids (including flavones, flavonols, flavan-3ols, anthocyanins, among others), phenolic acids (hydoxybenzoic or hydroxycinnamic) and tannins (condensed or hydrolysable). Acorns and other tree nuts are recognized for their high content of phenolic compounds which confer on them high antioxidant activity [5,6]. In our study, total phenolic compounds, flavonoids, and condensed tannins were determined by spectrophotometric techniques. The total phenolic content varied from 52.5 to 176.6 mg GAE/g, and flavonoid content was between 19 and 38 mg CE/g. These values are higher than those previously reported in Mexican *Q. virginiana* (total phenolics 29 mg GAE/g, flavonoids 13.9 mg CAE/g) from the same region [11], but in the range of values reported for various *Quercus* species, including *Q. ilex*, *Q. suber*, *Q. robur*, etc. [6,11]. It is worth mentioning that, although the phenolic compound content in the CHB population was significantly higher (2 to 3 times greater) than in the other three populations, the natural variability of these compounds is considerable and depends on both the species studied and the environmental and physiological conditions, including the existence of biotic or abiotic stress conditions [6]. For instance, a study on populations of *Quercus brantii* reported values ranging from 210 to 791 mg of tannic acid equivalents (TAE) per gram of dry extract, representing a nearly fourfold difference between populations [26]. Tannin content (condensed tannins) showed a variation of 0.01 to 0.26 mg CE/g, which is considerably lower than the tannin content reported in other *Quercus* species [5,6]. This can be viewed as a desirable trait, since high tannins are related to a bitter taste and are considered antinutritive agents because they form complexes with proteins and reduce their digestibility [27].

Actually, digestibility values differed among populations (68.5–74.4%), with the lowest value observed in CHA and the highest in CJA (Table 2). These results are similar to those found in Mexican *Q. virginiana* and Mediterranean *Q. ilex* [9,11], but lower than those determined from Mediterranean *Q. suber* and *Q. coccifera* [11]. Energy content also varied between populations (4853.8–4925.2 kcal/kg), with the lowest value observed in CHA and the highest in CJA (Table 2). These results were comparable to those determined in acorns from Spanish *Q. ilex* [9]. The differences in digestibility and energy values reflect the combined influence of macronutrient composition, particularly fat and carbohydrate content, as well as the presence of phenolic compounds and tannins. In this context, previous studies suggested that elevated tannin levels may reduce protein bioavailability and digestibility due to their ability to form insoluble complexes with proteins and digestive enzymes, underscoring their role as major antinutrients in acorns [9,27]. However, in our results, the content of total phenolic compounds was more related to digestibility than the content of condensed tannins, since the CJA population showed the highest digestibility and lowest content of total phenolics (Table 2). Moreover, the literature indicates that acorns may also contain phytates, oxalates, and certain fiber fractions that may limit mineral absorption or influence gastrointestinal function [4]. Therefore, future studies should incorporate a broader characterization of antinutrient compounds and evaluate different processing methods, such as soaking, heat treatments, or leaching, to improve digestibility and promote the safe and effective use of *Q. virginiana* acorns in food applications [4].

On the other hand, the antioxidant capacity of phenolic compounds is a desirable property. In our study, the antioxidant activity of acorns was determined by two techniques, FRAP and DPPH. FRAP-determined antioxidant activity (272 to 282 mmol TE/g) was similar to that found in acorns from *Q. virginiana* and *Q. suber*, but higher than that found for *Q. ilex* and much lower than that found for *Q. coccifera* [11]. These results were also consistent with those determined for *Q. robur* acorns [28]. DPPH-determined antioxidant activity had a small, non-significant variation among populations (112–114 mmol TE/g). This finding was also comparable to that observed in samples of *Q. robur* acorns, where DPPH values fluctuated within narrow intervals even when total phenolics differed substantially among the samples tested [28]. FRAP and DPPH techniques evaluate different mechanisms of antioxidant activity. The FRAP method measures the overall reducing power of polyphenols, whereas the DPPH assay assesses the hydrogen-donating capacity of specific compounds. As a result, DPPH values may exhibit small fluctuations despite substantial differences in phenol concentrations among samples [29], as was observed in the present study.

To further analyze the differences in acorn physicochemical composition, canonical discriminant analysis was performed. The analysis showed that the first two functions explained 97.8% of the total variation. The first function explained 87.9% of the total variation, and the variables that correlated positively with this function were carotenoids (r = 0.482), ash (r = 0.416), and total phenolic compounds (r = 0.236), but an inverse correlation was observed with the antioxidant activity determined by FRAP (r = −0.745) and fat content (r = −0.496). The second function represented 9.9% of the total variation, and the variables that correlated positively with this function were DPPH-determined antioxidant activity (r = 0.684), energy (r = 0.468), and the content of linoleic acid (r = 0.284), and palmitic acid (r = 0.116), but an inverse correlation was observed in the content of oleic acid (r = −0.120). To evaluate the classification performance and stability of the discriminant functions, we examined several diagnostic metrics. The original classification matrix indicated that 100.0% of cases were correctly classified, with 100.0% accuracy in each group. crossvalidated classification (Leave-one-out) yielded an overall accuracy of 91.7%, with groupwise values ranging from 66.7% to 100.0%, indicating that the model’s performance is robust despite the small sample size per group (n = 3).

The dispersion diagram of the two discriminant functions showed three well-established groups. One group was formed by the CHA (1) and CHB (2) populations, where both populations were distributed towards the left part of the diagram and were well separated from the group formed by the CJA (3) population. Finally, the group formed by the CJB (4) population was distributed towards the right part of the diagram (Figure 2). This group distribution closely resembles the one obtained from the morphological variables of acorns (Figure 1). This indicates that the two populations from the city of Chihuahua share more morphological and chemical traits (i.e., they are more homogeneous) than the populations from the city of Juarez. This could be related to genetic or environmental aspects and suggests an interesting link between morphometric characteristics and chemical composition, although further studies are needed to address these issues.

To understand if the variations in the content of nutrients and bioactive compounds in acorns of different populations could be related to geographic and climatic parameters (Table 4, in Materials and Methods), a Pearson correlation analysis was conducted (Table S2 in Supplementary Materials). Among nutrients, only ash contents (corresponding to minerals) were correlated to the geographic parameters (negative correlation with longitude and altitude). For fatty acids, the content of stearic acid was negatively correlated with temperature (mean annual maximal temperature) while linoleic acid was correlated with latitude and longitude. Fatty acid composition has been found to be correlated with the geographical origin of *Q. canariensis* acorns [30] and saturated fatty acid contents were positively correlated with soil salinity in *Q. virginiana* populations from various regions of China [14]. In our study, total phenolic compounds and condensed tannins were not correlated with any geographical or climatic variable; however, flavonoids and FRAP-determined antioxidant activity were positively correlated with average annual precipitation and negatively correlated with temperature (Table S2), showing that the antioxidant activity of acorns is closely related to the flavonoid content. In *Q. brantii* acorns, flavonoid content was also positively correlated with average annual precipitation, although the total phenolic compounds and tannin contents were positively correlated with average annual temperature [26], which is different from our results, especially considering the negative correlation we observed between flavonoids and temperature. In the same study by Ebrahimi et al. [26], total phenolic compounds and tannins were negatively correlated with altitude and longitude, and flavonoids were negatively correlated with altitude.

Although there are no studies that directly relate geographical and climatic factors to the nutritional composition and content of bioactive compounds in acorns from different populations of *Q. virginiana*, such associations have been documented in other species of the genus [9,12]. However, the relative contribution of the environmental factors in comparison to genetic and epigenetic characteristics remains unknown and is still speculative since direct experimental approaches are challenging [9]. In fact, a recent study concluded that the chemical composition of acorns from individual *Q. ilex* trees appeared to be more genetically than environmentally determined [9]. This would be especially true for the content of macronutrients (starch, lipid, protein) so it is possible that at least some of the differences found among the populations studied in the present work could be related to genetic variation. For example, CH populations could be both geographically and genetically closer to each other than to CJ populations. Moreover, physiological conditions of the trees and seeds (acorns) could also be involved in the chemical variability. On the other hand, the content of bioactive compounds, which are secondary metabolites, could be more dependent on the environmental factors. Phenolic compounds are produced in response to biotic and abiotic stress conditions [25], so their metabolism is highly dependent on factors such as water and nutrient availability, temperature, UV light exposure, etc. [31]. Although these environmental variables were not directly determined in the present paper, they can be affected by the different climatic and geographic conditions of the growing sites and may help to explain the variability in the content of bioactive compounds among populations, as has been suggested by other authors [14,26].

### 2.3. Polyphenolic Profile

Fourteen polyphenolic compounds were identified in *Q. virginiana* acorns from the four populations studied. Twelve were hydrolysable tannins (mostly ellagitannins); one hydroxycinnamic acid, and one flavonol were also found in some samples. Chlorogenic acid, ellagic acid and myricetin were quantified using authentic standards; surrogate standards (structurally similar) were used for the other compounds (Table 3). Spectral and chromatographic information used for the identification of the 14 compounds is summarized in Table S3 (Supplementary Materials).

The ellagitannin group was the most extensively represented polyphenolic group in all the populations, and the most abundant compounds belonged to this group (Table 3). Ellagitannins are a type of hydrolysable tannins, which are polyphenolic compounds found in various plants, including the genus *Quercus* [32]. Hydrolysable and condensed tannins are known for their ability to bind proteins, which can give certain plant materials an astringent taste, making them less palatable to animals and humans. They also have a role in plant defense mechanisms against herbivores and pathogens [33] and may possess several desirable bioactive properties, although ellagitannins and hydrolysable tannins in general have been scarcely studied. Ellagitannins can break down into ellagic acid, a compound with antioxidant properties, and potential anti-inflammatory and anticancer activity [4]. Actually, ellagic acid and its derivatives (pentoside, glucoside and methylated glucoside) were the most abundant phenolic compounds and were present in all the *Q. virginiana* populations. These compounds may be breakdown products of more complex ellagitannins, such as castalagin and punicalagin, which were also found in all populations, although at lower concentrations. Other ellagitannins such as davidiin and casuariin, were identified only in some populations; in fact, casuariin was found only in CHB samples. Gallotannins, the other type of hydrolysable tannins, which yield gallic acid upon hydrolysis, were the second most common group identified in *Q. virginiana* populations. Tetragalloylglucose could be quantified in the samples of two populations (CHA and CJA), and glucogallin could be identified but not quantified, only in CHA samples.

Studies on different *Quercus* species have consistently demonstrated that hydrolysable tannins (both gallotannins and ellagitannins) are the most representative phenolic compounds in acorns [7,34,35,36,37]. Hydrolysable tannins have also been identified in other organs of the *Quercus* trees [37,38]. Several of the compounds identified in the present study in *Q. virginiana* acorns, have also been identified in other species; these include ellagic acid [9,34], castalagin and punicalagin [6,35,36,39], and tetragalloylglucose [34,35]. It is worth mentioning that some species contain a majority of gallotannins while others contain more ellagitannins [7,35].

Regarding non-tannin compounds, two phenolics were detected, each restricted to a single population. Chlorogenic acid was exclusively identified and quantified in samples of the CJA population. This phenolic acid has been identified in acorns of various *Quercus* species [35,37] and is one of the most common phenolic compounds in the plant kingdom. Myricetin, a flavonol, was exclusively present in the samples of the CJB population, although it could not be quantified due to its low concentration. Myricetin has not been described in *Quercus* seeds, although it was detected in acorn leaves from *Q. liaotungensis* [40], and is widely distributed in fruits and vegetables.

## 3. Materials and Methods

### 3.1. Biological Material and Seed Collection

**Figure 3:**
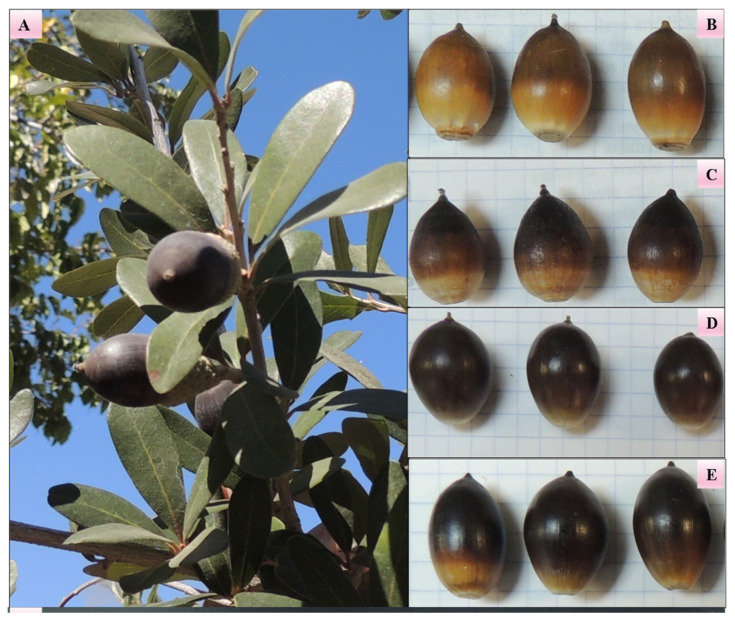
(**A**). Photography of a *Q. virginiana* tree (population CJB) showing the acorns in the tree crown. (**B**). Acorns of the CHA population. (**C**). Acorns of the CHB population. (**D**). Acorns of the CJA population. (**E**). Acorns of the CJB population.

*Q. virginiana* seeds were sampled from four populations located in two urban areas in the state of Chihuahua, Mexico. Two populations were selected in the city of Chihuahua, in the southern region of the state (CHA and CHB) (Figure 3B and 3C, respectively), and two populations were collected in the city of Juarez, in the northern region (CJA and CJB) (Figure 3D and 3E, respectively). For taxonomic identification, leaves, flowers, and acorns of *Q. virginiana* were collected and collated with the plant material deposited (RCD 7073 *Q. virginiana* Mill) in the herbarium of the plants of México located in the Autonomous University of Ciudad Juarez, Ciudad Juárez, Chihuahua, México. Table 4 shows the geographical and climatic characteristics of the provenances in which the biological material was collected. Climatic and geographical data were obtained from the database of the National Meteorological System of the National Water Commission, using historical data from 1950 to 2010.

Five healthy trees located 10 m from each other were selected to obtain the greatest variability in each of the sampled areas (Figure 3A). From each tree, only acorns distributed throughout the tree crown were collected, and the acorns that were located on the ground were discarded. The collected acorns were placed into an airtight polyethylene bag and immediately transported to the laboratory, where samples from each population were stored at 4 °C until the morphological and chemical analysis.

### 3.2. Acorn Morphology Analysis

The morphological characteristics of the seeds were determined according to the methodology described by Valero-Galván et al. [12]. Briefly, 60 individual unshelled acorns per population were photographed, and images were used to measure the length, width, perimeter, and area using the ImageJ software version 1.54k 15 September 2024 (ImageJ, Bethesda, MD, USA). The individual weights of the seeds were determined using an analytical balance (Mettler Toledo AJ150, Ciudad de México, México). To determine the seed coat and megagametophyte weights, the seed coat of the acorns was removed using a knife, and weights corresponding to the megagametophyte and coat were separately determined. Shelled seeds were photographed, and images were used to measure the length, width, area, and perimeter using the ImageJ software (ImageJ, Bethesda, MD, USA).

### 3.3. Preparation of Acorn Flour

Thirty seeds from each population were selected, and the seed coats were removed by making transversal and longitudinal cuts. Megagametophytes were ground using a blade mill (Moulinex, AD56 42, Lyon, France). For water content determination, triplicate subsamples (2 g) of the ground material were dried at 103 °C in a ventilated oven for 24 h, according to AOAC procedures [41]. The remaining material was dried at 45 °C for 48 h, homogenized using a Waring Blender (Waring Products, LB20E, New Hartford, CT, USA), and sieved through a 1 mm mesh to obtain a fine, homogeneous flour.

### 3.4. Determination of the Nutritional Composition

Homogeneous flour samples were analyzed in triplicate for each population by Near-Infrared Spectroscopy (NIRS) at the NIRS Service of the University of Córdoba (Spain), following the methodology of Valero-Galván et al. [12]. After spectral acquisition and processing, calibration equations previously developed for *Quercus ilex* subsp. *ballota* were applied to estimate protein, sugars, ash, starch, fiber, fat, linoleic acid (C18:2), palmitic acid (C16:0), stearic acid (C18:0), oleic acid (C18:1), digestibility, and energy. Total carbohydrates were calculated by difference [41].

### 3.5. Determination of Carotenoids

Carotenoids were determined according to the method proposed by Lichtenthaler [42], with minor modifications. Briefly, three independent replicates per population were prepared using 0.2 g of flour placed in a mortar. Next, 2 mL of 96% ethanol (DEQ, Naucalpan, Estado de México, Mexico) was added and mixed using a pestle. The homogenate was recovered into an assay tube using a micropipette, stirred at 500 rpm, and sonicated for 30 min at 4 °C in the dark. The extract was then centrifuged at 5000 rpm for 15 min at 4 °C, and the supernatant was transferred to a new tube. Finally, 300 µL of the extract was loaded into a 96-well microplate, and absorbance was measured at 470.0, 648.6, and 664.2 nm using an xMarkTM Absorbance Spectrophotometer with Microplate^®^ Manager 6.0 software (Bio-Rad, Hercules, CA, USA). Carotenoid concentration was calculated using the equations described by Lichtenthaler [42], and results were expressed as milligrams per gram of dry weight (mg/g DW).

### 3.6. Extraction of Phenolic Compounds

The phenolic extract was prepared following the methods proposed by Álvarez-Parrilla et al. [43]. Briefly, three independent replicates per population were prepared using 0.2 g of flour placed in a mortar. Then, 500 μL of 80% (*v*/*v*) methanol (JT Baker, Phillipsburg, NJ, USA) was added and mixed using a pestle. The homogenate was transferred to a tube, stirred at 500 rpm in the dark for 10 min, and sonicated at 4 °C in the dark for 30 min. The extract was centrifuged (Eppendorf, Enfield, CT, USA) at 3500 rpm for 15 min at 4 °C, and the supernatant was collected into a new tube. This procedure was repeated twice, and the supernatants were pooled to a final volume of 2 mL. Samples were stored at −20 °C until analysis.

### 3.7. Quantification of Total Phenolic Compounds, Flavonoids and Condensed Tannins

Total phenolic compounds and flavonoids were quantified following the methods proposed by Georgé et al. [44]. Briefly, 25 µL of the methanolic extract was mixed in a microplate well with 100 µL of 7.5% (*m*/*v*) sodium carbonate (ACS, Naucalpan, Estado de México, Mexico) and 125 µL of 10% (*v*/*v*) Folin–Ciocalteu reagent (ACS, Naucalpan, Estado de México, Mexico). The mixture was incubated at 30 °C in the dark for 15 min, and absorbance was measured at 760 nm. Quantification was performed using a calibration curve prepared with gallic acid (ACS, Naucalpan, Estado de México, Mexico). Results were expressed as milligrams of gallic acid equivalents per gram of dry weight (mg GAE/g DW). For flavonoid quantification, 310 µL of the methanolic extract were mixed with 125 µL of distilled water, 9.5 µL of 5% (*m*/*v*) sodium nitrite (ACS, Naucalpan, Estado de México, Mexico), 9.5 µL of 10% (*m*/*v*) aluminum chloride (ACS, Naucalpan, Estado de México, Mexico), and 125 µL of 0.5 M sodium hydroxide (ACS, Naucalpan, Estado de México, Mexico). The mixture was incubated at 27 °C in the dark for 30 min, and absorbance was measured at 510 nm. Quantification was performed using a calibration curve prepared with catechin (ACS, Naucalpan, Estado de México, Mexico). Results were expressed as milligrams of catechin equivalents per gram of dry weight (mg CE/g DW).

Condensed tannins were quantified using the p-dimethylaminocinnamaldehyde (DMAC) method. Briefly, 50 µL of the methanolic extract was mixed with 250 µL of 0.1% (*v*/*v*) DMAC reagent (N,N-dimethylacetamide, ACS, Naucalpan, Estado de México, Mexico) in 10% (*v*/*v*) HCl (ACS, Naucalpan, Estado de México, Mexico). The mixture was incubated at room temperature in the dark for 20 min, and absorbance was measured at 640 nm. Results were expressed as milligrams of catechin equivalents per gram of dry weight (mg CE/g DW). All determinations were performed in 96-well microplates (Costar^®^, Naucalpan, Estado de México, Mexico), and absorbance was measured using an xMarkTM Absorbance Spectrophotometer with Microplate^®^ Manager 6.0 software (Bio-Rad, Hercules, CA, USA).

### 3.8. Determination of Antioxidant Activity

Antioxidant activity was determined using the ferric reducing antioxidant power (FRAP) and 2,2-diphenyl-1-picrylhydrazyl (DPPH) assays, according to Moreno-Escamilla et al. [45]. For the DPPH assay, 25 µL of the methanolic extract was mixed with 200 µL of DPPH reagent (190 µM; ACS, Naucalpan, Estado de México, Mexico). The reaction mixture was incubated at 30 °C in the dark for 20 min, and absorbance was measured at 510 nm. For the FRAP assay, 25 µL of the methanolic extract was mixed with 180 µL of FRAP reagent (ACS, Naucalpan, Estado de México, Mexico). The reaction mixture was incubated at 30 °C in the dark for 20 min, and absorbance was measured at 595 nm. Quantification was performed using calibration curves prepared with Trolox (ACS, Naucalpan, Estado de México, Mexico) in the ranges 8–130 µM/mL (FRAP) and 25–400 µM/mL (DPPH). Results were expressed as mmol Trolox equivalents per gram of dry weight (mmol TE/g DW). Assays were conducted in triplicate in a 96-well microplate (Costar^®^, Naucalpan, Estado de México, Mexico), and absorbance was measured using an xMarkTM Absorbance Spectrophotometer with Microplate^®^ Manager 6.0 software (Bio-Rad, Hercules, CA, USA).

### 3.9. Identification of the Polyphenolic Profile by LC-MS-MS Analysis

Acorn flour was first defatted by mixing 10 g of flour with 100 mL of hexane and placing the mixture in an ultrasonic bath (Branson Ultrasonics, Brookfield, WI, USA) at 3500 Hz for 15 min, at room temperature. The mixture was centrifuged (15 min, 3500 rpm), the supernatant discarded, and the process repeated once more. The defatted flour was left overnight in an oven at 40 °C to eliminate hexane residue. Next, a sequential extraction was carried out, according to Pérez-Nájera et al. [46]. Briefly, 5 g of defatted flour were mixed with 125 mL of 70% (*v*:*v*) acetone in water, sonicated for 30 min (3500 Hz, RT) and centrifuged (15 min, 3500 rpm), recovering the supernatant. The procedure was repeated once more, and the two acetone supernatants were combined. A second extraction was carried out using 125 mL of 80% (*v*:*v*) methanol in acidified water (2%HCl, *v*:*v*), and the same conditions for sonication and centrifugation. Solvents were eliminated by rotary evaporation at 40 °C and the remaining aqueous extracts were combined and freeze-dried (Labconco, Kansas City, MO, USA) for 24 h to eliminate water.

The identification of phenolic compounds was carried out according to the method proposed by Muñoz-Bernal et al. [47]. Three independent samples of 0.03 g of acorn extract from each population were dissolved in 5 mL of 50% (*v*:*v*) acetonitrile in water. If needed, the samples were sonicated for 30 min at 4 °C in the dark until completely dissolved. Finally, 1 mL of each sample was filtered using a 2 µm Nylon syringe filter and placed in a 1.5 mL HPLC vial. Samples were injected (3 μL injection volume) into a UHPLC system (Infinity Series 1290, Aglient^®^ Technologies, Santa Clara, CA, USA) equipped with a C_18_ reversed-phase column (ZORBAX^®^, Santa Clara, CA, USA, 50 mm × 2.1 mm; 1.8 µm). The autosampler temperature was set to 20 °C, while the column thermostat temperature was 25 °C. Each sample was injected in triplicate. 0.1% (*v*:*v*) formic acid in water was used as solvent A and 100% acetonitrile was used as solvent B. The flow rate was set to 0.4 mL/min and the gradient program was as follows: 0–1 min 10% B, 1–4 min 30% B, 4–6 min 38% B, 6–8 min 60% B, 8–8.5 min 60% B, 8.5–9 min 10% B.

The mass spectrometer was equipped with electrospray ionization (Agilent 6530 Accurate Mass Q-TOF MS/MS, Aglient^®^ Technologies, Santa Clara, CA, USA) set in the negative ionization mode. Nitrogen was used as the drying gas at 340 °C and a flow rate of 13 L/min. The nebulizer pressure was set to 60 psi. The capillary voltage was set to 4000 V, fragmentation voltage was 175 V, and skimmer voltage was set to 65 V. Collision energy was set to 20 V. Masses were scanned between 100–1100 mass/charge ratio (*m*/*z*), and MS/MS masses were scanned between 50 and 1000 *m*/*z*. Data acquisition was performed in Auto MS/MS mode. Reference masses were: 119.036 and 966.0007. Quantification was performed according to the method described by Muñoz-Bernal et al. [48]. External calibration curves were constructed from the phenolic standards using the mass peak areas extracted from the ion chromatogram. Calibration curves were prepared by diluting the stock solutions in methanol (1 mg/mL of each compound). For compounds for which no standard was available, a calibration curve from structurally related compounds was used. The detection limit was calculated as three times the signal-to-noise ratio (S/N); while the quantification limit was calculated as 10 times S/N; both were calculated for each standard compound.

### 3.10. Statistical Analysis

The results are expressed as mean ± standard deviation of at least three independent experiments. Levene tests were performed to determine the equality of variance. If the samples presented equality of variances, a one-way analysis of variance (ANOVA) was performed, followed by a multiple comparison of means (Tukey) with a significance level of 0.05. Additionally, an analysis of canonical discriminant functions was performed to visualize the differences between species. The explained variance was analyzed, and the variables that contributed to the separation of species within the populations were identified using the canonical correlation coefficients of the structure matrix. All data were analyzed using the SPSS statistical software (IBM^®^, Armonk, NY, USA, version 15.0).

## 4. Conclusions

This study demonstrated marked morphological, nutritional and phytochemical variation among *Q. virginiana* populations in North Mexico. Differences in seed size and weight, nutrient profiles, and the abundance of phenolic compounds and antioxidant activity effectively distinguished the populations and reflected the influence of local climatic and geographic conditions. The identification of multiple polyphenolic compounds, mostly ellagitannins, highlights the biochemical complexity of the acorn and supports evidence of site-specific responses to environmental factors. These results provide a useful foundation for advancing the potential use of *Q. virginiana* acorns as a food ingredient, particularly considering their combination of favorable nutrients and bioactive compounds. Future research should evaluate the sensory properties and effect of processing on acorn products, as well as the ecological factors that shape chemical variability. Expanding sampling efforts and incorporating genetic analysis will also be essential to determine whether the observed differences arise from environmental influences or underlying genetic structure. A clearer understanding of these aspects will support the sustainable valorization and conservation of this species.

## Figures and Tables

**Table 1:** Acorn morphometry of four populations of *Q. virginiana*.

Morphometry Traits	Populations			
	CHA	CHB	CJA	CJB
Mass (g)	1.4 ± 0.3 ^a^	1.40 ± 0.2 ^a^	2.3 ± 0.6 ^d^	1.9 ± 0.5 ^c^
Length (cm)	2.0 ± 0.2 ^a^	2.0 ± 0.2 ^a^	2.4 ± 0.3 ^b^	2.3 ± 0.1 ^b^
Width (cm)	1.2 ± 0.1 ^a^	1.2 ± 0.1 ^a^	1.5 ± 0.1 ^b^	1.6 ± 0.1 ^c^
Area (cm)	1.9 ± 0.3 ^a^	1.9 ± 0.2 ^a^	2.7 ± 0.5 ^b^	2.8 ± 0.6 ^b^
Perimeter (cm)	5.1 ± 0.4 ^a^	5.1 ± 0.4 ^a^	6.0 ± 0.6 ^b^	6.0 ± 0.5 ^b^
Coat mass (g)	0.3 ± 0.0 ^a^	0.3 ± 0.0 ^a^	0.6 ± 0.2 ^c^	0.4 ± 0.1 ^b^
Megagametophyte mass (g)	1.0 ± 0.2 ^a^	1.0 ± 0.2 ^a^	1.7 ± 0.4 ^c^	1.4 ± 0.4 ^b^
Megagametophyte length (cm)	1.6 ± 0.1 ^a^	1.6 ± 0.3 ^a^	2.1 ± 0.3 ^c^	1.9 ± 0.1 ^b^
Megagametophyte width (cm)	1.0 ± 0.1 ^a^	1.1 ± 0.1 ^a^	1.3 ± 0.1 ^c^	1.2 ± 0.1 ^b^
Megagametophyte area (cm)	1.3 ± 0.2 ^a^	1.4 ± 0.2 ^a^	2.0 ± 0.5 ^c^	1.8 ± 0.3 ^b^
Megagametophyte perimeter (cm)	4.1 ± 0.4 ^a^	4.3 ± 0.5 ^a^	5.3 ± 0.6 ^c^	4.9 ± 0.4 ^b^

The descriptive statistics are presented in terms of the mean ± SD (n = 60 seeds per population). Mean values with the different letters differ significantly with *p* ≤ 0.05 according to the Tukey test.

**Table 2:** Acorns physicochemical composition of four populations of *Q. virginiana*.

Chemical Component	Population			
	CHA	CHB	CJA	CJB
Water (%)	71.6 ± 0.7 ^a^	71.7 ± 0.5 ^a^	72.1 ± 0.2 ^a^	73.1 ± 0.8 ^a^
Ash (%)	1.8 ± 0.0 ^a^	1.9 ± 0.0 ^a^	2.1 ± 0.0 ^b^	2.2 ± 0.0 ^c^
Protein (%)	4.3 ± 0.0 ^a^	5.3 ± 0.0 ^b^	7.0 ± 0.06 ^d^	5.7 ± 0.0 ^c^
Carbohydrates (%)	82.5 ± 0.5 ^d^	80.8 ± 0.10 ^c^	78.1 ± 0.1 ^a^	79.3 ± 0.1 ^b^
Starch (%)	58.8 ± 0.1 ^d^	57.3 ± 0.0 ^c^	53.4 ± 0.1 ^a^	56.03 ± 0.1 ^b^
Sugar (%)	8.1 ± 0.1 ^a^	12.1 ± 0.4 ^b^	13.3 ± 0.1 ^c^	13.8 ± 0.2 ^c^
Fiber (%)	2.4 ± 0.0 ^a^	2.4 ± 0.0 ^a^	2.5 ± 0.0 ^b^	2.4 ± 0.0 ^a^
Fat (%)	11.3 ± 0 ^a^	12.0 ± 0.0 ^b^	12.7 ± 0.0 ^c^	12.7 ± 0.0 ^c^
Oleic (%)	70.8 ± 0.5 ^d^	65.0 ± 0.3 ^c^	60.3 ± 0.7 ^b^	58.3 ± 0.5 ^a^
Palmitic (%)	14.6 ± 0.1 ^a^	15.9 ± 0.2 ^b^	17.7 ± 0.1 ^c^	19.1 ± 0.2 ^d^
Linoleic (%)	12.4 ± 0.1 ^a^	13.0 ± 0.0 ^b^	17.3 ± 0.0 ^c^	19.1 ± 0.1 ^d^
Stearic (%)	4.0 ± 0.1 ^c^	4.3 ± 0.0 ^d^	3.7 ± 0.0 ^b^	3.0 ± 0.1 ^a^
Digestibility (%)	68.5 ± 0.1 ^a^	73.7 ± 0.4 ^b^	74.4 ± 0.5 ^b^	73.9 ± 0.3 ^b^
Energy (kcal/100 g)	4853.8 ± 1.3 ^a^	4882.7 ± 1.6 ^b^	4925.2 ± 2.0 ^d^	4896.2 ± 2.3 ^c^
Carotenoids (mg/g)	5.1 ± 2.2 ^b^	1.1 ± 0.6 ^a^	3.2 ± 2.1 ^ab^	2.1 ± 0.6 ^ab^
Phenolic compounds ^*^	78.73 ± 7.99 ^c^	176.6 ± 12.7 ^d^	63.7 ± 5.4 ^b^	52.5 ± 2.4 ^a^
Flavonoids ^**^	29.03 ± 0.01 ^b^	38.03 ± 0.03 ^c^	19.01 ± 0.03 ^a^	20.02 ± 0.01 ^a^
Condensed tannins ^**^	0.07 ± 0.0 ^b^	0.01 ± 0.0 ^a^	0.07 ± 0.0 ^ab^	0.26 ± 0.01 ^c^
FRAP ^***^	278.0 ± 2 ^b^	282.0 ± 4 ^b^	272.0 ± 2 ^a^	270.0 ± 2 ^a^
DPPH ^***^	114.0 ± 0.0 ^a^	112.0 ± 2 ^a^	114.0 ± 2 ^a^	114. ± 2 ^a^

Data are expressed on a dry weight basis, mean ± SD of three replicas. Mean values with different letters differ significantly with *p* ≤ 0.05 according to the Tukey test. ^*^ mg gallic acid equivalents (GAE)/g; ^**^ mg catechin equivalents (CE)/g; ^***^ mmol TROLOX equivalents (TE)/g.

**Table 3:** Polyphenolic compounds identified and quantified (mg/g of extract) in *Quercus virginiana* acorns from four populations of the north of Mexico.

Polyphenolic Compound		Populations			Type
	CHA	CHB	CJA	CJB	
Chlorogenic acid ^1^	NI	NI	0.25 ± 0.03	NI	Hydroxycinnamic acids
Castalagin ^2^	0.16 ± 0.01	0.16 ± 0.01	˂LOQ	˂LOQ	Ellagitannins
Casuariin ^2^	NI	0.19 ± 0.02	NI	NI	Ellagitannins
Davidiin ^2^	0.20 ± 0.00	NI	0.17 ± 0.02	˂LOQ	Ellagitannins
Ellagic acid ^3^	1.99 ± 0.06	1.79 ± 0.18	1.95 ± 0.03	1.22 ± 0.02	Ellagitannins
Ellagic acid glucoside ^3^	0.46 ± 0.02	0.46 ± 0.04	0.50 ± 0.03	0.48 ± 0.01	Ellagitannins
Ellagic acid pentoside ^3^	0.77 ± 0.06	0.72 ± 0.16	0.97 ± 0.37	1.02 ± 0.01	Ellagitannins
Hexahydroxydiphenoyl-D-glucose ^3^	0.31 ± 0.00	0.28 ± 0.01	NI	0.27 ± 0.00	Ellagitannins
Methyl ellagic acid pentoside ^3^	0.59 ± 0.00	0.54 ± 0.03	0.66 ± 0.02	0.47 ± 0.00	Ellagitannins
Punicalagin ^2^	0.2 ± 0.01	˂LOQ	0.21 ± 0.03	0.2 ± 0.02	Ellagitannins
Myricetin ^4^	NI	NI	NI	˂LOQ	Flavonols
Glucogallin ^5^	˂LOQ	NI	NI	NI	Gallotannins
Pentagalloylglucose ^5^	˂LOQ	NI	˂LOQ	NI	Gallotannins
Tetragalloylglucose ^5^	0.24 ± 0.00	NI	0.28 ± 0.00	˂LOQ	Gallotannins

NI: not identified; NQ: identified but not quantified; ˂LOQ: identified under the quantification limit. Quantification was performed with the following standards: ^1^ chlorogenic acid, ^2^ corilagin, ^3^ ellagic acid, ^4^ myricetin, ^5^ gallic acid.

**Table 4:** Geographical and climatic data of the collection sites.

Characteristics	Population			
	CHA	CHB	CJA	CJB
Latitude	28°44′17.9′′ N	28°43′07′′ N	31°41′33.9′′ N	31°44′46′′ N
Longitude	106°07′54′′ W	106°06′59.3′′ W	106°22′52.4′′ W	106°26′37′′ W
Altitude (m)	1430	1420	1120	1125
MmminT (°C) ^a^	10.3	9.9	11.5	11.0
MmAT (°C) ^b^	18.3	17.7	19.2	19.0
MmmaxT (°C) ^c^	26.3	25.5	27.0	28.0
AP (mm) ^d^	368.3	442.8	142.9	160.0

^a^ Mean monthly minimum temperature; ^b^ Mean monthly annual temperature; ^c^ Mean monthly maximum temperature; ^d^ Annual precipitation.

## Data Availability

The data presented in this study are available on request from the corresponding author.

## Supplementary Materials

The following supporting information can be downloaded at: https://www.mdpi.com/article/10.3390/molecules31091438/s1, Table S1. Pearson correlation of the climatic and geographical variables and the seed morphometric traits; Table S2. Pearson correlation of the climatic and geographical variables and the contents of nutrients and bioactive compounds; Table S3. Spectral information of phenolic compounds identified in the *Quercus* samples.
