# Supplementary material for: Potential of North American Acorns as an Underutilized Food Source: Morphology, Nutritional Composition and Content of Bioactive Compounds in Quercus virginiana Acorns of Different Natural Populations
Source: Molecules. 2026 Apr 27;31(9):1438. doi: 10.3390/molecules31091438 (PMC13165087; doi:10.3390/molecules31091438)
Supplement: Supplementary file 1 [file molecules-31-01438-s001.zip › molecules-4177962-supplementary.pdf]

SUPPLEMENTARY MATERIAL

Potential of North American acorns as an underutilized food source: morphology, nutritional composition and content of bioactive compounds in *Quercus virginiana* acorns of different natural populations

José Valero-Galván[1], Oscar A. Muñoz-Bernal[1,2], Raquel González-Fernández[1], Jesús Jorrín Novo[3], Laura A. De La Rosa[1*]

[1]Departamento de Ciencias Químico-Biológicas, Instituto de Ciencias Biomédicas, Universidad Autónoma de Ciudad Juárez, Av. Plutarco Elías Calles #1210 Fovissste Chamizal, Ciudad Juárez, Chihuahua, C.P. 32310, México.

[2]Secretaria de Ciencia, Humanidades, Tecnología e Innovación (SECIHTI), Insurgentes Sur 1582, Col. Crédito Constructor, Alcaldía Benito Juárez, Ciudad de México C.P. 03940, México.

[3]Agroforestry and Plant Biochemistry, Proteomics, and Systems Biology, Department of Biochemistry and Molecular Biology, ETSAM, University of Cordoba, UCO-CeiA3,14014 Cordoba, Spain.

***Corresponding author: ldelaros@uacj.mx**

Table S1. Pearson correlation of the climatic and geographical variables and the seed morphometric traits

	Mass (g)	Length (cm)	Width (cm)	Area (cm)	Perimeter (cm)	Coat mass (g)	Mm (g)[5]	Ml (cm)[6]	Mw (cm)[7]	Ma (cm)[8]	Mp (cm)[9]
Latitude	0.922	.97(*)	.98(*)	.99(**)	1.00(**)	.783	.928	.916	.889	.95(*)	.939
Longitude	-0.859	-.938	-.99(**)	-.99(**)	-.98(*)	-.687	-.867	-.850	-.824	-.908	-.882
Altitude (m)	-0.931	-.98(*)	-.97(*)	-.99(**)	-1.00(**)	-.800	-.937	-.928	-.905	-.96(*)	-.95(*)
MmminT (ºC)[1]	0.97(*)	.96(*)	.855	.904	.930	.888	.97(*)	.927	.850	.937	.929
MmAT (ºC)[2]	0.904	.932	.884	.913	.926	.773	.907	.845	.753	.876	.856
MmmaxT (ºC)[3]	0.663	.777	.929	.899	.870	.428	.674	.618	.559	.703	.661
AP (mm)[4]	-0.924	-.96(*)	-.950	-.97(*)	-.97(*)	-.784	-.929	-.888	-.825	-.926	-.907

[1]Mean monthly minimum temperature; [2]Mean monthly annual temperature; [3]Mean monthly maximum temperature; [4]Annual precipitation; [5]Megagametophyte mass; [6]Megagametophyte length; [7]Megagametophyte width; [8]Megagametophyte area; [9]Megagametophyte perimeter; * The correlation is significant at the 0.05 level (bilateral); ** The correlation is significant at the 0.01 level (bilateral).

Table S2. Pearson correlation of the climatic and geographical variables and the contents of nutrients and bioactive compounds

	Latitude	Longitude	Altitude	MmminT (ºC)[1]	MmAT (ºC)[2]	MmmaxT (ºC)[3]	AP (mm)[4]
Water	,767	-,879	-,795	,561	,650	,908	-,743
Ash	,934	-,963 (*)	-,951 (*)	,768	,772	,843	-,872
Protein	,823	-,706	-,814	,797	,668	,402	-,731
Fat	,901	-,878	-,914	,744	,686	,656	-,798
Carbohydrates	-,904	,831	,905	-,820	-,727	-,566	,811
Sugar	,763	-,751	-,784	,550	,479	,507	-,622
Fiber	,620	-,447	-,587	,771	,632	,188	-,603
Digestibility	,636	-,590	-,655	,438	,324	,290	-,471
Energy	,845	-,741	-,840	,792	,671	,443	-,746
Starch	-,864	,751	,853	-,856	-,740	-,470	,791
Palmitic	,901	-,942	-,922	,711	,721	,828	-,831
Stearic	-,802	,905	,818	-,675	-,786	-,987 (**)	,832
Oleic	-,882	,889	,901	-,691	-,657	-,702	,780
Linoleic	,958 (*)	-,991 (**)	-,970	,823	,846	,909	-,925
Carotenoids	-,133	,155	,171	,152	,231	,060	-,057
Phenols	-,704	,736	,689	-,795	-,896	-,862	,830
Flavonoids	-,912	,906	,900	-,954 (*)	-,996 (**)	-,901	,975 (*)
Tannins	,625	-,772	-,650	,465	,610	,921	-,662
FRAP	-,935	,965 (*)	,934	-,903	-,962 (*)	-,969 (*)	,976 (*)
DPPH	,580	-,600	-,558	,724	,827	,754	-,730

[1]Mean monthly minimum temperature; [2]Mean monthly annual temperature; [3]Mean monthly maximum temperature; [4]Annual precipitation; * The correlation is significant at the 0.05 level (bilateral); ** The correlation is significant at the 0.01 level (bilateral).

Table S3. Spectral information of phenolic compounds identified in the *Quercus* samples

Compound	R.T. (min)	Formula	[M-H]	Experimental mass (Da)	Theorical mass (Da)	Diff (ppm)	Fragments
Chlorogenic Acid	0.527	$C_{16} H_{18} O_9$	353.0867	354.094	354.0951	-3.06	**353.0860**; 291.0139
Castalagin	0.516	$C_{41} H_{26} O_{26}$	933.0636	934.0715	934.0712	0.31	**933.0641**;
Casuariin	0.535	$C_{34} H_{24} O_{22}$	783.0688	784.0762	784.0759	0.37	**783.0700**; 633.0728
Davidiin	3.477	$C_{41} H_{30} O_{26}$	937.094	938.1014	938.1025	-1.22	**937.0931**; 867.0878;
Ellagic acid	3.842	$C_{14} H_6 O_8$	300.9984	302.0056	302.0063	-2.09	**300.9976**; 165.9058; 61.9871
Ellagic acid glucoside	3.021	$C_{20} H_{16} O_{13}$	463.0519	464.0592	464.0591	0.15	**463.0518**; 371.980
Ellagic acid pentoside	3.481	$C_{19} H_{14} O_{12}$	433.0408	434.0481	434.0485	-0.98	**433.0408**; 403.1623
Glucogallin	0.411	$C_{13} H_{16} O_{10}$	331.0676	332.0742	332.0744	-0.59	**331.0694**;
Hexahydroxydiphenoyl-D-glucose	0.398	$C_{20} H_{18} O_{14}$	481.0635	482.0708	482.0697	2.41	**481.0651**;
Methyl ellagic acid pentoside	3.819	$C_{20} H_{16} O_{12}$	447.0569	448.0644	448.0642	0.42	**447.0570**; 433.0408
Punicalagin	3.823	$C_{48} H_{28} O_{30}$	1083.0594	1084.0668	1084.0665	0.25	**1083.0593**;
Myricetin	4.736	$C_{15} H_{10} O_8$	317.0287	318.0362	318.0376	-4.36	**317.0292**;
Pentagalloylglucose	4.177	$C_{41} H_{32} O_{26}$	939.1111	940.117	940.1182	-1.27	**939.1109**;
Tetragalloylglucose	3.706	$C_{34} H_{28} O_{22}$	787.0993	788.1064	788.1072	-1.02	**787.0999**;

Fragments in bold indicate the most intense fragment identified.
